# Round the Hospitals

**Published:** 1919-01-18

**Authors:** 


### ROUND THE HOSPITALS.

A very long list of awards in connection with the Order of the British Empire was issued on January 11. None has been more richly earned by great powers nobly used for the public good than the appointment of Miss Swift, R.R.C., Matron-in-Chief of the B.R.C.S., Dame Grand Cross of the Order of the British Empire. Hers is one of the creative minds which can, from a wealth of detail amassed with laborious care, strike out the hidden idea. Like her successors at Guy's Hospital, the work she has done among the auxiliary hospitals is fully known only to those behind the scenes. Dame Sarah Swift is a power on the Council of the College of Nursing, and her influence for good in the profession ripens as time goes on. The appointment of Miss Rachel Eleanor Crowdy, R.R.C., Principal Commandant of V.A.D.s in France, to be Dame Commander of the Order, will give great pleasure to a wide circle. Among those who are made Commanders and Officers of the Order we note the names of a large number of county organisers, donors and commandants of military hospitals, and secretaries of different departments of war work in this country. The lists, touching as they do a mere fringe of the great body of voluntary war work, give a forcible impression of the hitherto almost untapped energies of the public. If this source can only be turned to account for national purposes in the reconstruction ahead the country ought to be able to blow the slums out of existence and banish for ever the abuses which sap the life of the community.

---

The London Centre of the College of Nursing has been too busy stamping out influenza to have had much energy left for its own interest. Like many of the newly-formed provincial centres, it found itself swamped almost as soon as founded by urgent public duties, and has carried on only with extreme

**ROUND THE HOSPITALS**—*(continued)*.

difficulty in a time when every member was doing the work of two or three other people besides her own. The new premises, 7 Henrietta Street, have, however, been got into order through it all, and they will be inaugurated by a social gathering to take place on January 23, from 7 to 8 p.m., before the State Registration meeting is opened. It is probable that a large accession of members will pour in on that occasion, for the club-rooms are so central, and so much needed in this Harley Street neighbourhood, that they have only to be known to be appreciated.

---

The Demobilisation Bureau of the College is getting into its stride and proving very popular with members. Much trouble has been taken to obtain early notice of public posts likely to become available for nurses, and the authorities are in touch with the Ministry of Reconstruction and the Ministry of Labour, and hope soon to be in a position to furnish information in regard to the demand for and supply of nurses. In regard to the prompt filling of vacant posts, we understand that the well-known vacillation of nurses in applying for work, and the swift and often unaccountable changes of mind they are apt to experience in the process of getting "suited," form an embarrassing feature in Bureau routine. The educative side of the Bureau seems to us, however, of far more importance than its function as intermediary.

---

"Nurses feel that there has been quite enough talking," said Miss Gill, R.R.C., at a recent meeting of the Association for the Promotion of the Registration of Nurses in Scotland, presided over by Professor Glaister. Miss Gill considered that the College of Nursing is "the most democratic and progressive movement," and she urged adherence to the registration provided by the College rather than to that of the Central Committee. Those present at the Edinburgh meeting sympathised with Miss Gill's point of view, and the Chairman's proposition was unanimously carried, that the Association, having considered its relation to the Central Committee in London for State Registration of Nurses and the College of Nursing (Limited), did now withdraw from the Central Committee and would use every effort to promote the College of Nursing Bill. Dr. R. M'Kenzie Johnston said that the Scotch Association had been sitting on the fence quite long enough, and had undoubtedly at last come to a right conclusion.

---

Miss Cowlin's visit to France was extremely timely, coming hard as it did on the demobilisation of the B.R.C.S., but before that of the Military Nursing Services. During her fortnight on the other side of the Channel she visited Boulogne, Abbeville, Amiens, Trouville, Havres, Le Treport. Though for three days at Christmas no formal work could be attempted, she held eighteen meetings during the fortnight she spent in France, sometimes three in one day. She met everywhere a most cordial reception and an eager desire to learn about the College of Nursing, and her tour is already beginning to bear fruit in the form of numerous applications for membership from nursing sisters returning home. Many meetings, some formal and some informal, for discussion among nurses have been held in the various hospitals in France since her departure to discuss the new ideas opened up, and we believe all must have felt indebted to the College of Nursing for thus anticipating their desire for information on the burning topics of the hour at home.

---

We congratulate the General Infirmary, Stockport, on its appointment to the post of matron of Miss Florence Goodacre, who for some years has been assistant matron at the Preston Royal Infirmary. Our experience of Miss Goodacre's work justifies our confidence and the high opinion we have formed of her. She was trained at the Royal Infirmary, Manchester, and at Leicester Isolation Hospital, later becoming theatre charge-nurse and ward sister at the former Institution. She has served in the Territorial Force Nursing Service in English hospitals, and abroad as theatre sister, home sister, and night superintendent, and holds the certificate of the Incorporated Society of Trained Masseuses. There were sixty-one applicants for the matronship of Stockport Infirmary, and Miss Goodacre was one of three candidates finally selected to come before the Board, the others being: Miss Bailey, assistant matron, Royal Infirmary, Derby, and Miss Rastall, matron of Princess Alice Memorial Hospital, Eastbourne.

---

The authorities of the Glasgow Royal Infirmary are now able to point, with justifiable pride, to the fact that their nurses are better paid than in any other institution in that town. They have recently granted a substantial increase in salary to all ranks. To nurses in training an increase of £7 per annum in each of the four years during training raises salaries to £19, £23, £27, and £32. Fifth-year nurses receive an addition of £10, making the salary £45; sixth-year nurses, with a similar addition, now receive £50; seventh-year nurses, with an addition of £13, receive £55; and the maximum is reached in the eighth year at £60. It is an interesting feature of these alterations that the probationer with previous fever training is now to receive remuneration in proportion to her increased value to the institution, and commences at £23, proceeding in subsequent years to £27, £32, £39, and £50. These increases are in addition to certain bonuses which have been granted for the past two years, and date as from October 1 last.

---

At a meeting held on December 30 at the Town Hall, Rye, it was unanimously, and with much enthusiasm, decided to build a cottage hospital as a war memorial for Rye, Winchelsea and district. Dr. Bulton stated there was no possibility of dealing with emergency operations in Rye, and no means of getting a nurse who could remain with a case. Should any one be found dying near Rye there was no place to which such a case could be removed. Great difficulties also arose in regard to acute cases,

**ROUND THE HOSPITALS—(*continued*).**

such as pneumonia, because at the Hastings Hospital the beds were wanted for urgent surgical cases, and they could seldom accept medical patients. Dr. Bulton was able to tell the meeting that of the £6,000 required to build and partially endow a hospital with from six to ten beds, £5,000 had already been promised and £50 a year towards upkeep; also that he had the promise of an admirable site. Several of those present spoke warmly of the help accorded to the town and district by the Hastings (East Sussex) Hospital, 480 cases having been received there from Rye and district for free treatment during the past four years. It seems highly probable that the possession of a cottage hospital of their own will stimulate Rye residents to a deeper interest in the care of the sick, and thus actually increase the volume of support accorded to the Hastings Hospital, which has never reached its proper proportions.

The dangers of sleep shortage were very acutely discussed by Miss May Smith, lecturer in psychology, at a recent meeting of the Association of University Women Teachers at King's College. Miss Smith has been experimenting on her own person and that of others on the effects of doing without sleep for an abnormal length of time. She finds that the subject passes through two distinct phases, and nurses who have been compelled in emergencies or epidemics to do without sleep confirm her observations. At first there is a sense of stimulus; the work is done even better than usual. Next the body attempts to make good its losses. There is a general loss of accuracy, a weakening in the power of inhibition, a loss of concentration and retentiveness. This is attended, however, by no sense of tiredness. The subject believes herself to be in her best form, though in such a phase she may receive an order, reply to it, and forget it instantly, mis-read a prescription, or administer a double dose without perceiving the error. This is the really dangerous stage of sleeplessness, part of the faculties being already closed down while the rest are awake and lively. Few will deny the conclusion arrived at—that it is extravagant to demand good work from the fatigued.

One of the best, because one of the most natural, ways of keeping babies alive is by bringing mothers back to the practice of breast-feeding. In no direction has Dr. Truby King been more successful than in his dealings with the difficulties which have led doctors and midwives, in a large proportion of cases, to acquiesce in the rejection of this mode of feeding by the mothers under their care. The courses of instruction in mothercraft now available at the Institute, 29-31 Trebovir Road, Earl's Court, deal mainly, though not exclusively, with this subject, and are being attended by nurses and midwives from different parts of the kingdom, who will, it is hoped, carry back the teaching to many important centres. It has been already demonstrated that the methods developed by the Babies of the Empire Society are successful in very many cases where the power of breast-feeding has been lost.

The process of demobilising the Nursing Services is likely to be considerably accelerated by the appointment of the new Committee under the Ministry of Labour in consultation with the Admiralty and the War Office. With all their modern improved machinery, neither the Navy nor the Army are exactly renowned for their ability to consider employment questions from the point of view of the employee, and it will be much for the good of the Nursing Services that representatives of the hospitals and other civilian bodies employing nurses should meet the representatives of the naval and military authorities to advise in questions of priority of release for nursing sisters. Institutions desirous of securing release for nurses formerly in their employ are to communicate with the Nurses' Demobilisation and Resettlement Committee, Ministry of Labour, 16 Curzon Street, Mayfair, W. 1. Members of the Nursing Services must on their side fill in a civil employment form, and when the "marriage" of these respective forms has been effected, that in which the institution claims the nurse being matched with that in which the nurse sues to return to it, something will have been accomplished. But the hour of release is not yet, we fear.

We are asked to state that the spring course arranged by the Royal Sanitary Institute for Women Health Visitors, Tuberculosis Visitors, School nurses, and school teachers commences February 21, at 6 p.m. The fee for the course is £1 11s. 6d. A supplementary course on Child-Welfare is given at a similar fee, or the two in combination can be taken at a reduction of 10s. 6d. Students will attend a course of six infant consultations, under the direction of Dr. G. Eric C. Pritchard.

The American Red Cross Society has sent a further donation of 50,000 dollars to Mrs. Laurie, the honorary treasurer of the Scottish Women's Hospital, Edinburgh. This is the third instalment of the splendid new war grant of 150,000 dollars made to the Scottish Women's Hospital at the instance of Miss Kathleen Burke. The work in Serbia still demands the utmost energy and devotion from the unit in charge of the sick and wounded. The S.W.H. have recently taken over a hospital with 500 beds at Vranja, near Uskub, where British soldiers are nursed in wards adjoining those for numerous other nationalities. There are no doctors in the town; very little clothing or food can be procured, for the Germans have swept the country bare, and to crown all Spanish influenza is raging.

Tea adulteration often used to be one of the housekeeper's main anxieties, for in every stage of its journey, from the plant to the tea-cup, it furnished opportunities. Thanks, however, to the tests carried out by the Government chemist, the bogey of adulterated tea has now practically vanished from the store-room. The number of samples officially examined at the port of entry in the year ending March 31 was 8,556. Of these 1,485, representing

**ROUND THE HOSPITALS—(*continued*).**

over 100,000 lb. of tea, were condemned as containing sand or other foreign matter, and 101 other samples were reported unfit for human consumption. The total amount of tea imported is 311,000,000 lb., so that the results of the analysis may be considered very satisfactory. Grumblers do not always realise that the Government retail price of tea at 2s. 8d. the lb. includes 1s. per lb. duty. Until the existing stocks, bought at war rates and carried subject to war charges, are cleared out the price will not be lowered.

Mr. Hoover, having made a personal visit to the various European countries appealing for food, has reached the conclusion that 1,400,000 tons of foodstuffs will be required to supplement the food supplies in Europe until next harvest and keep the populations from starvation. The cost is estimated at about £20,000,000. The mortality among the children is said to be appalling. The situation is specially serious in Finland, the Baltic States, Serbia, Jugo-Slavia, Tirol, Poland, Rumania, Bulgaria, Armenia, and Czecho-Slovakia. The facts ought to be kept incessantly in mind; every crust of bread wasted is taken from the common stock of supplies which furnishes relief to these unhappy peoples.

The Ministry of Reconstruction have lately issued a valuable memorandum on "Subsidiary Health and Kindred Services for Women," by Miss A. M. Anderson, C.B.E., in which the value of female orderlies and portresses as described above is brought to notice. The list of occupations open to women in the campaign against infant mortality and disease includes twelve headings, and exposes many comparatively new methods of attacking social evils. It may be obtained price 3d. from H.M. Stationery Office, Imperial House, Kingsway, W.C.2. It is admirably put together and should be widely read.

The inquest on the V.A.D. worker Miss Boshell, whose unhappy story we recently referred to, came to an unsatisfactory end on January 10. The jury returned a verdict to the effect that the deceased died from the effects of veronal poisoning, but how administered there was not sufficient evidence to show. The Coroner expressed himself baffled by the mysterious and conflicting evidence, and openly stated that he believed all the facts in the case had not yet come to light. It transpired that the deceased lady had been privately married in France.

The remarkable decrease in pauperism revealed by the latest available figures is a source of deep thankfulness to all whose labours lead them among the sick and aged poor. Probably no duty is more painful than that which devolves on district nurses, often enough of getting their patients "put away," to use their pathetic euphemism for removal to the workhouse. To learn that there were 16,688 fewer indoor cases in 1917 than in 1916, and that the decrease has continued to operate steadily this year up to the last set of figures issued in September, may perhaps strike some readers as a rather dull and dry bit of statistics. But when translated by a touch of imagination into reality it points to a sum total of home hearths kept burning and hope struggling successfully against despair such as might gladden every heart did men but heed the mute dramas in progress only round the corner. Under the new *régime* of the Health Ministry which looms ahead we look for great improvement on the old, but foremost in our expectations is the restoration of respect—may we not even say in reverence?—of the treatment of our aged poor.

